# Novel developments in angiogenesis cancer therapy

**DOI:** 10.3747/co.v16i3.444

**Published:** 2009-05

**Authors:** H.W. Hirte

**Keywords:** Angiogenesis, targeted therapy, combination therapy

## Abstract

A greater understanding of the factors and pathways controlling angiogenesis in tumours has allowed the development of a number of agents that selectively inhibit the pathways mediating that activity. The results to date of clinical trials with such agents and future research opportunities are reviewed.

## 1. INTRODUCTION

An exciting new area in cancer research and treatment involves the study of a class of agents called angiogenesis inhibitors. These agents block the process of angiogenesis—the development of new blood vessels. Angiogenesis is one of the hallmark features that tumours require for both growth and metastasis^1^. For a tumour to grow beyond 1–2 mm in size, it needs to induce the formation of new blood vessels to supply its nutritional and other needs^2^. This is the process that anti-angiogenic agents target. Cutting off the supply of nutrients and oxygen to a tumour will prevent its continued growth and spread to other parts of the body.

In most situations, endothelial cells lie dormant. But when needed, short bursts of blood vessel growth occur in localized parts of tissues. New vessel growth is tightly controlled by a finely tuned balance between factors that activate endothelial cell growth and those that inhibit it. In an adult, physiologic angiogenesis occurs in the ovaries, uterus, and placenta. Pathologic angiogenesis occurs in the process of wound healing, and in a number of disease states, including cancer.

A number of proteins are known to activate endothelial cell growth and movement. These include vascular endothelial growth factor (vegf), acidic and basic fibroblast growth factors, angiogenin, epidermal growth factor, scatter factor, placental growth factor, interleukin-8, and tumour necrosis factor α^3^. Known naturally occurring inhibitors of angiogenesis include angiostatin, endostatin, interferons, platelet factor 4, thrombospondin, transforming growth factor β, 16-kDa fragment of prolactin, and tissue inhibitor of metalloproteinase-1, -2, and -3^3^. In addition, the processes of both angiogenesis and metastasis require matrix metalloproteinases—enzymes that break down the surrounding tissue (the extracellular matrix) during blood vessel growth and tumour invasion.

At a critical point in the growth of a tumour, when the “angiogenic switch” is flipped to shift the tumour microenvironment to a pro-angiogenic state, the tumour sends out signals to the nearby endothelial cells to activate new blood vessel growth.^3^ The vegf family of growth factors and its receptors constitute the most important signalling pathways in tumour angiogenesis^4^. Angiogenesis is also related to metastasis, assisting in the spread of a tumour to various parts of the body. It is generally true that tumours with higher densities of blood vessels are more likely to spread and to have poorer clinical outcomes. The shedding of large numbers of tumour cells from the primary tumour may not begin until after the tumour has a network of blood vessels.

## 2. TARGETS AND AGENTS

The possibility of inhibiting angiogenesis as a potential therapeutic intervention in cancer treatment was first proposed by Folkman^5^. A number of anti-angiogenic drugs are now either licensed or in clinical trials (Table i). In general, four strategies are being used by investigators to design anti-angiogenesis agents^2^:

- Blocking the ability of the endothelial cells to break down the surrounding matrix
- Inhibiting normal endothelial cells directly
- Blocking factors that stimulate angiogenesis
- Blocking the action of integrin, a molecule on the endothelial cell surface

### 2.1 Results of Clinical Trials to Date

**FIGURE 1:**
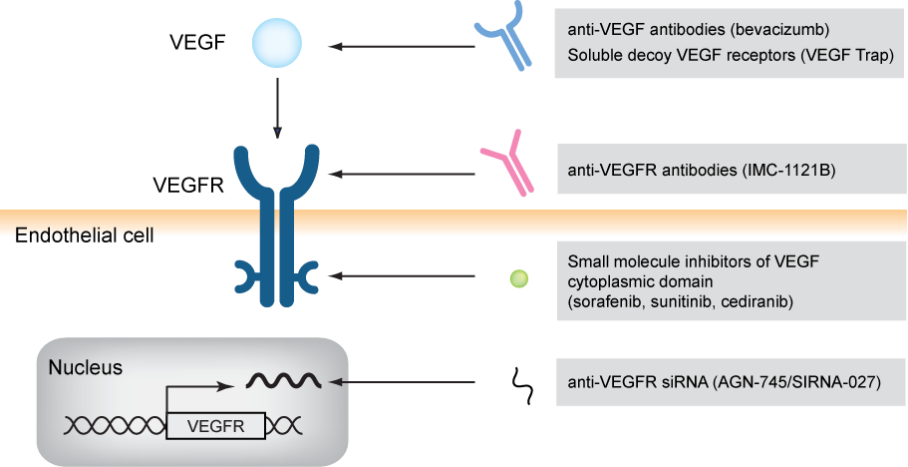
*A number of agents have been developed that target the vascular endothelial growth factor (vegf) pathway. These include antibodies and soluble receptors that specifically bind to* *vegf, antibodies that prevent* *vegf* *receptor (vegfr) activation, small-molecule inhibitors that block the tyrosine kinase activity of the intracellular domain of the* *vegf* *receptor, and small interfering* *rnas (sirnas) that inhibit the messenger* *rna* *for* *vegfr*.

All currently approved anti-angiogenic drugs (Figure 1) block either vegf or vegf tyrosine kinase receptors (vegfrs). These agents have been used either as single agents or in combination with conventional chemotherapeutic regimens. To date, the survival benefits of anti-angiogenic agents have been modest, but this modest success has led to interest in developing more effective ways to combine these agents with standard cytotoxic chemotherapies and with other agents targeting specific signalling pathways in tumour cells.

Single-agent activity has been demonstrated with sunitinib, which inhibits the vegfr tyrosine kinase in advanced renal cancer^6^, and sorafenib, which inhibits the vegfr tyrosine kinase and also has Raf kinase inhibitory activity in renal cancer and hepatoma^7^,^8^. Bevacizumab, the first anti-angiogenic agent approved by the U.S. Food and Drug Administration, significantly increased overall survival or progression-free survival in patients with metastatic colorectal cancer, non-small-cell lung cancer, and breast cancer when given in combination with conventional chemotherapeutic agents^9^–^11^.

Inhibiting the messenger rna (mrna) for either angiogenic growth factors or their receptors is another approach that could be used to inhibit factors that stimulate angiogenesis. Although no such agents have yet been tested in the cancer setting, one such agent, AGN-027, which is a small interfering rna (sirna) directed against vegfr-1 mrna, has shown promise in ocular neovascularization and may be promising in the oncology setting^12^.

Some of the differences between standard chemotherapy and anti-angiogenesis therapy result from the targeting by angiogenesis inhibitors of dividing endothelial cells rather than tumour cells. Anti-angiogenic drugs are not as likely to cause symptoms such as bone marrow suppression, gastrointestinal symptoms, or hair loss, which are characteristic of standard chemotherapy treatments. Also, because anti-angiogenic drugs may not necessarily kill tumours, but rather hold them in check indefinitely, the endpoint of early clinical trials may be different from those used in standard therapies. Rather than look for tumour response only, an evaluation of increases in survival or time to disease progression may be appropriate.

### 2.2 Toxicities

Anti-vegf therapy can lead to specific—and sometimes unexpected—toxicities such as hypertension, proteinuria, bowel perforation, hemorrhage, and arteriothrombotic events. It is thought that the hypertension associated with anti-vegf therapy is a result of inhibition of endothelial cell–derived nitric oxide, which is a pathway known to be mediated by vegfr2 activation. However, the basis of other toxicities, such as bowel perforation, remains unclear^13^.

Agents such as bevacizumab or aflibercept have exquisite specificity for their target (vegf); agents such as sorafenib and sunitinib, which block the intracellular tyrosine kinase domain of the vegf receptor, have a broader spectrum of activity and inhibitory effects on a number of receptors such as platelet-derived growth factor receptor and c-Kit in addition to vegfr. Broader specificity may be important in preventing the development of resistance to inhibition of angiogenesis; however, broader specificity is also associated with a broader range of potential toxicities such as fatigue and skin and gastrointestinal toxicities^14^.

### 2.3 Mechanisms of Resistance

Drug resistance is a major problem with chemotherapy agents. Most cancer cells are genetically unstable, more prone to mutations, and therefore likely to produce drug-resistant cells. Because anti-angiogenic drugs target normal endothelial cells, which are genetically stable, it was initially hoped that resistance to these agents might not develop. However, despite major advances in the clinical development of vegf-targeted therapy, tumours in most patients are inherently resistant. When a response is obtained, the benefit of vegf inhibitors is transient, generally lasting only weeks to months. Therefore, nearly all tumours are either inherently resistant or develop acquired resistance to agents targeting the vegf/vegfr pathway^15^.

The emergent mechanisms of resistance include revascularization because of upregulation of alternative pro-angiogenic signals; protection of the tumour vasculature by recruitment of pro-angiogenic inflammatory cells or by an increase in protective pericyte coverage; accentuated invasiveness of tumour cells into local tissue to co-opt normal vasculature; and increased metastatic seeding and tumour cell growth in lymph nodes and distant organs^16^.

### 2.4 Combinations with Standard Therapy or Other Targeted Agents

Anti-angiogenesis therapy may prove useful in combination with therapy directly aimed at tumour cells. Because each therapy is aimed at a different cellular target, the hope is that the combination will prove more effective. Because anti-angiogenics are generally cytostatic rather than cytoreductive, combinations involving conventional cytotoxic chemotherapies may be useful for maximizing therapeutic activity.

A number of hypotheses have been proposed with respect to the mechanistic basis by which anti-angiogenic drugs enhance the effects of chemotherapy. Anti-angiogenics targeting the vegf pathway reduce the hyperpermeable nature of the tumour vasculature such that a transient reduction will occur in the high tumour interstitial fluid pressures. In addition, the chaotic and dysfunctional tumour-associated vasculature can be transiently normalized, allowing for better delivery of chemotherapeutic agents to the tumour environment. This transient “window” of vascular normalization and decrease in interstitial pressure may allow for an increase in the concentration of chemotherapy agents delivered to the tumour^17^.

By understanding the pathways that become upregulated during the development of resistance to anti-angiogenics, it may possible to combine anti-vegf inhibition strategies with approaches that inhibit these resistance pathways to achieve more effective inhibition of angiogenesis^15^.

### 2.5 Surrogate Markers of Anti-angiogenic Activity

An ideal surrogate marker could be used to guide the clinical development of the anti-angiogenics and to select the patients most likely to benefit from this approach. A number of such potential surrogate markers have been assessed, including circulating proteins (vegf or other pro-angiogenic growth factors), soluble vegfr, and various adhesion molecules associated with vascular endothelial cells that can be released into the circulation^15^. Measuring the number of circulating cells thought to be relevant to angiogenesis, including circulating endothelial cells and circulating endothelial progenitor cells, is also a possibility^18^. Noninvasive imaging by dynamic contrast-enhanced magnetic resonance, computed tomography, or high-frequency ultrasound can also be used to assess blood flow or vascular permeability^18^. Thus far, however, none of these approaches has been validated in the clinical setting.

## 3. SUMMARY

Angiogenesis is a process crucial to the growth and metastasis of cancer. Agents such as inhibitors of the vegf pathway have shown promising activity as single agents and in combination with standard chemotherapy agents. However, inherent or acquired resistance to these agents eventually develops. Understanding these mechanisms of resistance and validating surrogate makers of anti-angiogenic activity will be key to developing more effective anti-angiogenic therapies in the future.

## Figures and Tables

**TABLE I:** Anti-angiogenic agents

*Target*	*Agent*	*Description*
vegf ligand	Bevacizumab (Avastin^a^)	Humanized anti-vegfa monoclonal antibody
	Aflibercept (vegf Trap^b^)	Fusion protein of vegfr-1, vegfr-2, and immunoglobulin G1 Fc fragment
vegf receptor		
Extracellular domain	IMC-1121B	Human anti–vegfr-2 monoclonal antibody
Cytoplasmic domain	AEE788	vegfr-2 and egfr inhibitor
	Axitinib (AG-013736)	vegfr-1,2 and pdgfr inhibitor
	BMS-582664	vegfr-2 and fgfr inhibitor
	Motesanib (AMG-706)	vegfr-1,2,3; pdgfrb; and c-Kit inhibitor
	OSI-930	vegfr-2 and c-Kit inhibitor
	Pazopanib (GW-786034)	vegfr-2 inhibitor
	Tandutinib (MLN-518)	Flt-3, pdgfr, c-Kit inhibitor
	Vatalanib (PTK787)	vegfr-1,2,3 and pdgfrb inhibitor
	Sorafenib (BAY 43–9006)	b-Raf; vegfr-2,3; pdgfrb; Flt-3; and c-Kit inhibitor
	Sunitinib (SU11248)	vegfr-1,2; pdgfra, pdgfrb; and c-Kit inhibitor
	Cediranib (AZD2171)	vegfr-1,2,3; pdgfrb; and c-Kit inhibitor
	Vandetanib (ZD6474)	vegfr-2,3 and egfr inhibitor
	XL-184	vegfr-2, Met, c-Kit, Flt-3, and Tie2 inhibitor
	XL-999	vegfr-1,2,3; fgfr; pdgfr; and Flt-3 inhibitor
Endogenous inhibitors	Angiostatin	Cleavage fragment of plasminogen
	Endostatin	Cleavage fragment of collagen xviii
	Thrombospondin 1	Extracellular glycoprotein
Other	EMD 121974 (Cilengitide^c^)	α_v_β_3_ Integrin receptor inhibitor
	ATN-161	α_v_β_1_ Integrin receptor inhibitor
	Volociximab	α_v_β_1_ Integrin receptor inhibitor
	Vitaxin	Human monoclonal antibody to α_v_β_3_ integrin receptor
	AMG-386	Angiopoietin inhibitor
	Thalidomide	Immunomodulatory agent
	AGN-745/SIRNA027	sirna inhibiting vegfr-1 mrna

^a^ Genentech, San Francisco, CA, U.S.A.

^b^ Regeneron, Tarrytown, NY, U.S.A.

^c^ Merck and Co., Whitehouse Station, NJ, U.S.A.

vegf = vascular endothelial growth factor; vegfr = vascular endothelial growth factor receptor; egfr = epidermal growth factor receptor; pdgfr[a,b] = platelet-derived growth factor receptor [α, β]; fgfr = fibroblast growth factor receptor.

## References

[1] Hanahan D, Weinberg RA (2000). The hallmarks of cancer. Cell.

[2] Folkman J (2007). Angiogenesis: an organizing principle for drug discovery?. Nat Rev Drug Discov.

[3] Hanahan D, Folkman J (1996). Patterns and emerging mechanisms of the angiogenic switch during tumorigenesis. Cell.

[4] Ferrara N, Gerber HP, Lecouter J (2003). The biology of vegf and its receptors. Nat Med.

[5] Folkman J (1971). Tumor angiogenesis: therapeutic implications. N Engl J Med.

[6] Motzer RJ, Hutson TE, Tomczak P (2007). Sunitinib versus interferon alfa in metastatic renal-cell carcinoma. N Engl J Med.

[7] Escudier B, Eisen T, Stadler WM (2007). on behalf of the target Study Group. Sorafenib in advanced clear-cell renal-cell carcinoma. N Engl J Med.

[8] Llovet JM, Ricci S, Mazzaferro V (2008). on behalf of the sharp Investigators Study Group. Sorafenib in advanced hepatocellular carcinoma. N Engl J Med.

[9] Hurwitz H, Fehrenbacher L, Novotny W (2004). Bevacizumab plus irinotecan, fluorouracil, and leucovorin for metastatic colorectal cancer. N Engl J Med.

[10] Sandler A, Gray R, Perry MC (2006). Paclitaxel–carboplatin alone or with bevacizumab for non-small-cell lung cancer. N Engl J Med.

[11] Miller K, Wang M, Gralow J (2007). Paclitaxel plus bevacizumab versus paclitaxel alone for metastatic breast cancer. N Engl J Med.

[12] Shen J, Samul R, Silva RL (2006). Suppression of ocular neovascularization with sirna targeting vegf receptor 1. Gene Ther.

[13] Hurwitz H, Saini S (2006). Bevacizumab in the treatment of metastatic colorectal cancer: safety profile and management of adverse events. Semin Oncol.

[14] Demetri GD, van Oosterom AT, Garrett CR (2006). Efficacy and safety of sunitinib in patients with advanced gastrointestinal stromal tumour after failure of imatinib: a randomised controlled trial. Lancet.

[15] Ellis LM, Hicklin DJ (2008). Pathways mediating resistance to vascular endothelial growth factor-targeted therapy. Clin Cancer Res.

[16] Bergers G, Hanahan D (2008). Modes of resistance to anti-angiogenic therapy. Nat Rev Cancer.

[17] Jain RK (2005). Normalization of tumor vasculature: an emerging concept in antiangiogenic therapy. Science.

[18] Hicklin DJ, Ellis LM (2005). Role of the vascular endothelial growth factor pathway in tumor growth and angiogenesis. J Clin Oncol.

